# Low-density lipoprotein cholesterol and atrial fibrillation; A Mendelian randomization study using UK-Biobank data

**DOI:** 10.23889/ijpds.v1i1.322

**Published:** 2017-04-19

**Authors:** Michail Katsoulis, Spiros Denaxas, Riyaz Patel, Harry Hemingway

**Affiliations:** 1 University College London; 2 Farr Institute of Health Informatics Research

## Objectives

We used data from UK-Biobank that were linked with Hospital Episode Statistics and Office for National Statistics to assess the relationship between low-density lipoprotein cholesterol (LDL-C) and atrial fibrillation (AF). In this study, we applied Mendelian randomization in order to find out whether there is a causal effect of LDL-C to AF.

## Approach

We used data from the UK Biobank (~500,000 subjects) which is linked with electronic health records. At baseline (2006-2010), participants from across the UK took part in this project. They have undergone measures, provided blood, urine and saliva samples for future analysis, detailed information about themselves and agreed to have their health followed. Information in relation to the development of atrial fibrillation was derived from a) the enrollment of the participants (self-reported events), b) their hospitalization before and after their recruitment to UK-Biobank (confirmed events from Hospital Episode Statistics) and c) the death certificates [confirmed events from Office for National Statistics]. We also used genetic data from the analyses of the participants' blood sample that have been stored. We used Mendelian randomization to capture the effect of LDL-C to AF. As instruments, we used a genetic predisposition risk score (GPRs) for LDL-C, which was created as a weighted sum of the 18 most significant SNPs related to LDL-C, as there were documented in Global Lipid Consortium, in 18 out of 22 chromosomes. We ran a logistic regression model, using AF as outcome and GPRs as exposure. 

## Results

Our final sample consisted of 144,092 individuals, for which we have valid information for their genetic data. The AF cases in this sample were 3207, most of which were identified from Hospital Episode Statistics (hospitalization of the participants). From the Mendelian randomization study, from our preliminary results, we found a weak positive relationship between GPRs and AF, when we did not adjust for any covariate [OR per one unit increase of GPRs=1.08, 95% CI= (0.95-1.22)] and results remained practically the same when we adjusted for age and sex [OR=1.09, 95% CI= (0.96-1.24 )]. 

## Conclusions

We observed a weak positive association between LDL-C and AF in this study. This is the first Mendelian randomization approach that focuses on this relationship. More Mendelian randomization studies should be performed in order to identify the causal effect of LDL-C to AF. The use of electronic health records will facilitate the conduction of similar studies.

